# Utilizing typical developmental trajectories to reflect brain abnormalities in autism spectrum disorder

**DOI:** 10.1093/psyrad/kkae024

**Published:** 2025-01-23

**Authors:** Long-Biao Cui, Xian-Yang Wang, Hua-Ning Wang

**Affiliations:** Shaanxi Provincial Key Laboratory of Clinic Genetics, Fourth Military Medical University, Xi'an 710032, China; Schizophrenia Imaging Laboratory, Department of Psychiatry, Xijing 986 Hospital, Fourth Military Medical University, Xi'an 710054, China; Department of Radiology, The First Affiliated Hospital of Xi'an Jiaotong University, Xi'an 710061, China; Schizophrenia Imaging Laboratory, Department of Psychiatry, Xijing 986 Hospital, Fourth Military Medical University, Xi'an 710054, China; Department of Psychiatry, Xijing Hospital, Fourth Military Military Medical University, Xi'an 710032, China

Autism spectrum disorder (ASD) is an overwhelmingly complex and heterogeneous neurodevelopmental disorder characterized by challenges in social interaction and communication, alongside restricted and repetitive behaviors and interests. The etiopathogenesis of ASD remains incompletely understood, presenting challenges to effective diagnosis and treatment (Lord *et al*., 2018). Currently, the diagnosis of ASD continues to be guided primarily by the Diagnostic and Statistical Manual of Mental Disorders and International Classification of Diseases.

Neuroimaging techniques such as magnetic resonance imaging (MRI), positron emission tomography, and single-positron emission tomography have been extensively employed to elucidate the neurophysiological mechanisms underlying ASD. These techniques facilitate non-invasive examination of brain structure and function, providing insights into the anatomical, functional, and neurochemical or molecular changes in ASD (Li *et al*., 2021; Shan *et al*., 2024). For instance, a previous review article identified increased parieto-temporal lobe, and cerebellar volumes as consistent MRI findings in autism, suggesting a neural network abnormality (Brambilla *et al*., 2003). Findings from a voxel-based MRI study indicated reduced grey matter and increased cerebrospinal fluid volume in children with autism, with implications for social brain connectivity (McAlonan *et al*., 2005). Also, advanced MRI techniques have been instrumental in exploring ASD's pathophysiology, with a focus on synaptic pruning, gyrification, and neurotransmitter imbalances (Rafiee *et al*., 2022). Nogay and Adeli utilized deep learning for multiple classification of ASD in MRI scans, achieving improved diagnostic accuracy rates (Nogay and Adeli, 2024). A previous study also aimed to link the neuroimaging results with genetic level (Neklyudova *et al*., 2021). For ASD with exceptional arithmetic ability, neuroimaging study have implicated a network of brain regions in the processing of numerical information and arithmetic skills, including the frontal and parietal cortex (Vogel and De Smedt, 2021). These regions are known to be involved in the representation of numerical magnitude and in the execution of arithmetic operations. However, despite this potential, findings from neuroimaging studies in ASD remain inconclusive. Variability in study findings and the complexity of ASD as a heterogeneous condition presenting significant challenges (Duan and Chen, 2022; Huang *et al*., 2023; Li *et al*., 2022), thereby limiting the integration of neuroimaging findings into clinical practice (Lord *et al*., 2020).

In the journal, Zhao *et al*. presented a case of an autistic child with exceptional arithmetic abilities (Zhao *et al*., 2024). The authors conducted comprehensive behavioral assessments on the 8-year-old particpant, diagnosing autism with co-occurring intellectual and language disorders. They then investigated the brain development of the subject based on MRI. Gaussian process regression was used to model gray matter volume (GMV) developmental trajectories from age 3 to 12, using data from 110 typically developing controls. Deviation scores were calculated based on the relative deviation of the particpant's GMV from the normative developmental trajectories. These scores characterize the differences between autistic and typically developing children. This report underscores neurodiversity in ASD, offering insights into brain development differences. The findings and methodology of this report are intriguing, and the report should be commended for its succinct methodology but with clinical and translational implications.

However, there are also several concerns when interpretating these findings. The original study's findings, while intriguing, require a more rigorous statistical approach and clearer presentation to strengthen the conclusions drawn. For example, the statistical method used to compare GMV values of the case and controls could be further illustrated. Also, the explanation and description of their figure 1 could be extended. There could be further discussion as to why only one standard deviation was shown and why GMV values and percentages shifted across age axes. Additionally, there could be broader interpretation of brain regions involved. The original study's interpretation of GMV differences across a wide range of brain regions may not sufficiently distinguish the specific contributions of these areas to arithmetic abilities. The observed differences in GMV could be associated with a broader spectrum of cognitive and developmental factors, not limited to arithmetic skills. For instance, lower GMV in the left inferior frontal gyrus, bilateral temporo-parietal cortex, and left ventral occipital-temporal cortex could be accounting for mental retardation or speech deficits. Furthermore, a more targeted analysis comparing children with ASD who exhibit exceptional arithmetic skills to those without such skills could provide additional insights into the neurobiological underpinnings of these abilities. The implications for personalized diagnosis and treatment, as suggested in our commentary, should be grounded in a more robust understanding of the neurodevelopmental correlates of exceptional abilities in ASD.

“Autism spectrum” denotes the continuum of severity and manifestations of autistic traits, underscoring the extensive variability in symptoms, skills, and levels of impairment or disability observed in individuals with autism (Grzadzinski *et al*., 2013). This variability reflects the heterogeneity of the condition and emphasizes the necessity of personalized approaches in diagnosis and intervention. Normative models are powerful emerging statistical techniques in neuroimaging research. Normative models function similarly to pediatric growth charts, offering individual-level statistical inferences by measuring deviations from the normative range (Marquand *et al*., 2019). Traditional case-control analyses compare patients with controls at a group level, assuming a clear distinction between patients and control groups (Wolfers *et al*., 2020). Such analyses tend to obscure inter-individual differences. By contrast, normative models offer individualized and parameterized metrics for each patient, using normative ranges to reflect and capitalize on inter-individual differences. These models provide essential benchmarks for understanding typical brain development and identifying deviations in neurological and psychiatric disorders, advancing the field toward personalized medicine.

Longitudinal and cross-sectional neuroimaging studies have revealed atypical developmental trajectories in individuals with ASD (Guo *et al*., 2017; Li *et al*., 2021). Zhao *et al*. utilized structural brain images of 110 typically developing children to establish typical gray matter developmental trajectories. By comparing the brain neuroimaging data of the autistic particpant to normative trajectories, was able to identify specific regions where gray matter development deviated from typical patterns. These deviations provide insights into the neural mechanisms underlying ASD, highlighting regions that may contribute to the observed behavioral and cognitive differences. This study represents an important role in capturing inter-individual differences in neuroimaging, moving towards the development of individualized diagnosis in ASD. However, considerable gaps remain in the realization of precision medicine for ASD.

Therefore, it is necessary to further develop the normative model in application to clinical practice. For example, in this study, it allows for individual-level statistical inferences by measuring deviations from the normative range. This could be used to identify specific areas where the child's development deviates from typical patterns. Moreover, by identifying these deviations, clinicians can design personalized intervention plans that target the child's unique strengths and challenges. For example, if a child with autism shows atypical development in the neural networks associated with social interaction, targeted social skills training could be incorporated into their treatment plan. The normative model can also be used to monitor a child's progress over time, comparing subsequent assessments to the normative trajectory to evaluate the effectiveness of interventions and make adjustments as needed.

In summary, the impact of neuroimaging on clinical practice and public health remains limited. Few results and models from neuroimaging have been incorporated into clinical settings, and neuroimaging has yet to significantly influence diagnostic or therapeutic approaches in ASD (Woo *et al*., 2017). Several advancements are essential for neuroimaging to substantially affect clinical practice, public health, and personalized diagnosis and treatment (Fan *et al*., 2023; Wang *et al*., 2024). Future research should focus on enhancing the robustness and generalizability of normative models, incorporating larger and more diverse datasets, and developing methods to integrate these models into clinical workflows.
